# Mini review on skin biopsy: traditional and modern techniques

**DOI:** 10.3389/fmed.2025.1476685

**Published:** 2025-03-05

**Authors:** Nasar Alwahaibi, Maryam Alwahaibi

**Affiliations:** ^1^Biomedical Science, College of Medicine and Health Science, Sultan Qaboos University, Muscat, Oman; ^2^Biomedical Science, Sultan Qaboos University, Muscat, Oman

**Keywords:** skin biopsy, light microscopy, immunofluorescence, artificial intelligence, convolutional neural networks

## Abstract

The incidence of skin cancer continues to rise due to increased sun exposure and tanning habits, requiring early detection and treatment for favorable outcomes. Skin biopsy is an important diagnostic tool in dermatology and pathology, as it provides a valuable understanding of various skin diseases. Proper handling of skin biopsy specimens is vital to ensure accurate histopathological assessment. Still, the use of light microscopy and immunofluorescence provides a comprehensive approach to evaluating skin biopsy specimens, with each contributing unique information to aid in accurate diagnosis and management. This review highlights the evolution of skin biopsy practices, from traditional techniques to advanced methods incorporating artificial intelligence (AI) and convolutional neural networks. AI technologies enhance diagnostic accuracy and efficiency, aiding in the rapid analysis of skin lesions and biopsies. Despite challenges such as the need for extensively annotated datasets and ethical considerations, AI shows promise in dermatological diagnostics. The future of skin biopsy lies in minimally invasive techniques, liquid biopsies, and integrated pharmacogenomics for personalized medicine.

## Introduction

Skin cancer is one of the most common types of cancer globally, affecting millions of people each year. The primary types are basal cell carcinoma (BCC), squamous cell carcinoma (SCC), and melanoma, with BCC being the most frequent (1). In 2020, non-melanoma skin cancer (NMSC), after lung and prostate cancers, was the third most frequently diagnosed cancer in males worldwide. In fact, NMSC was the first and most common cancer in Northern America, Australia, and New Zealand (2). The rate of skin cancer is increasing because of more sun exposure and tanning habits. Detecting and treating skin cancer early is essential for a positive outcome, but there are obstacles to diagnosis and healthcare accessibility. Raising public awareness and promoting preventive actions, like applying sunscreen and limiting sun exposure, are critical to lowering the risk of skin cancer (3, 4).

Skin biopsies are crucial diagnostic procedures in dermatology, providing valuable information about various skin conditions. Skin biopsy is valuable in distinguishing between different types of skin diseases, including but not limited to bullous pemphigoid, dermatitis herpetiformis, pemphigus vulgaris, epidermolysis bullosa simplex, discoid lupus erythematosus, systemic lupus erythematosus, erythema multiforme, lichen planus actinicus, leukocytoclastic vasculitis, vasculitis, urticarial vasculitis, polymorphous light eruption, demodex follicularis, porphyria, thrombotic thrombocytopenia, vitiligo, and psoriasis. Furthermore, it helps in diagnosing spongiotic patterns such as spongiotic dermatitis, pompholyx, and pityriasis. Recent advances in skin biopsy techniques for diagnosing alopecia have improved precision in identifying underlying causes, particularly in distinguishing between scarring and non-scarring types (5). Key techniques include the use of punch biopsy, ideally taken at a 4 μm thickness to ensure deeper analysis and assessment across all follicle levels (6). Both vertical and horizontal sectioning are commonly used to evaluate different layers of hair structure and associated pathologies (7, 8). Findings such as follicular miniaturization, perifollicular fibrosis, and the ratio of growth phase to resting phase hairs help in diagnosing conditions such as androgenetic alopecia, alopecia areata, and scarring alopecia (9).

A typical skin biopsy involves taking a small tissue sample from the skin and examining it under a microscope to identify abnormalities and diagnose different skin diseases (10). This method provides detailed information about the tissue’s structure and any pathological changes. The combined use of light microscopy (LM) and immunofluorescence (IF) provides a full approach to diagnosing skin biopsies.

LM, through the use of mainly hematoxylin and eosin (H&E), offers detailed views of cellular architecture, which facilitates the identification of general pathological changes. IF techniques, which involve the use of antibodies tagged with fluorescent dyes to detect specific antigens in the tissue, are useful for diagnosing autoimmune and inflammatory diseases, such as lupus erythematosus and pemphigoid, by revealing the presence and distribution of immune deposits in the skin. Combining LM and IF enhances diagnostic accuracy and provides a comprehensive evaluation of skin biopsy specimens (11).

The literature lacks a review that bridges traditional biopsy techniques with modern advancements such as AI and convolutional neural networks (CNNs). This gap leaves a need for a resource that highlights the progression from traditional to modern techniques and discusses their combined potential to enhance diagnostic accuracy. As the incidence of skin cancer continues to rise, there is a pressing need for improved diagnostic tools and techniques. Traditional skin biopsy methods, while effective, can be further enhanced by incorporating modern technologies such as AI and CNNs (12). These technologies promise to increase diagnostic accuracy and efficiency, addressing some limitations of current practices. This review highlights the evolution of skin biopsy practices, from traditional techniques to advanced methods incorporating AI and CNNs.

### Histopathological processing of skin biopsies

After obtaining informed consent, the area of the skin to be biopsied is cleaned with an antiseptic solution, and using a dissecting microscope to look for gross indications such as blisters or vesicles, a fresh skin biopsy is bisected into two pieces: one half for light microscopy and the other half for immunofluorescence (13). For light microscopy, the skin biopsy portion is fixed in 10% neutral buffered formalin, processed for histology, sectioned at 3 μm thickness, and stained using the hematoxylin and eosin method. For immunofluorescence, the skin biopsy portion is snap-frozen in liquid nitrogen, sectioned at 5 μm thickness, fixed in cold acetone, and stained with fluorescence isothiocyanate-conjugated antibodies.

### Immunofluorescence (IF)

IF is a powerful technique used in conjunction with skin biopsy to diagnose various skin diseases. In dermatology, IF is particularly useful for diagnosing autoimmune blistering diseases and other dermatoses (14). When combined with a skin biopsy, IF provides detailed information about the presence and distribution of immune components in the skin (15). Direct IF (DIF) is the most commonly used method for skin biopsies. DIF demonstrates IgG, IgA, and IgM immunoglobulins, and C3 complement in skin biopsies (16). For example, DIF shows IgG and C3 deposits in the epidermis in pemphigus vulgaris, reveals linear IgG and C3 deposits along the basement membrane zone in Bullous Pemphigoid, demonstrates granular IgA deposits at the dermal-epidermal junction in dermatitis herpetiformis, and shows a full band of IgG, IgM, IgA, and C3 at the dermo-epidermal junction in lupus erythematosus (17, 18).

### Skin frozen section

The frozen section procedure for skin biopsy is a quick, intraoperative diagnostic method, often used in surgeries for skin cancer, such as Mohs micrographic surgery (MMS) (19). After the tissue sample is collected, it is rapidly frozen in a cryostat, allowing the tissue structure to be preserved temporarily without formalin fixation. A measure of 5–6 μm thickness is then cut using a cryostat and stained with hematoxylin and eosin, for microscopic examination. Then, the pathologist assesses the tissue’s cellular structures and determines whether cancer cells are present at the margins, which guides the surgeon on whether further excision is needed (20).

### Types of skin biopsy

**Figure 1:**
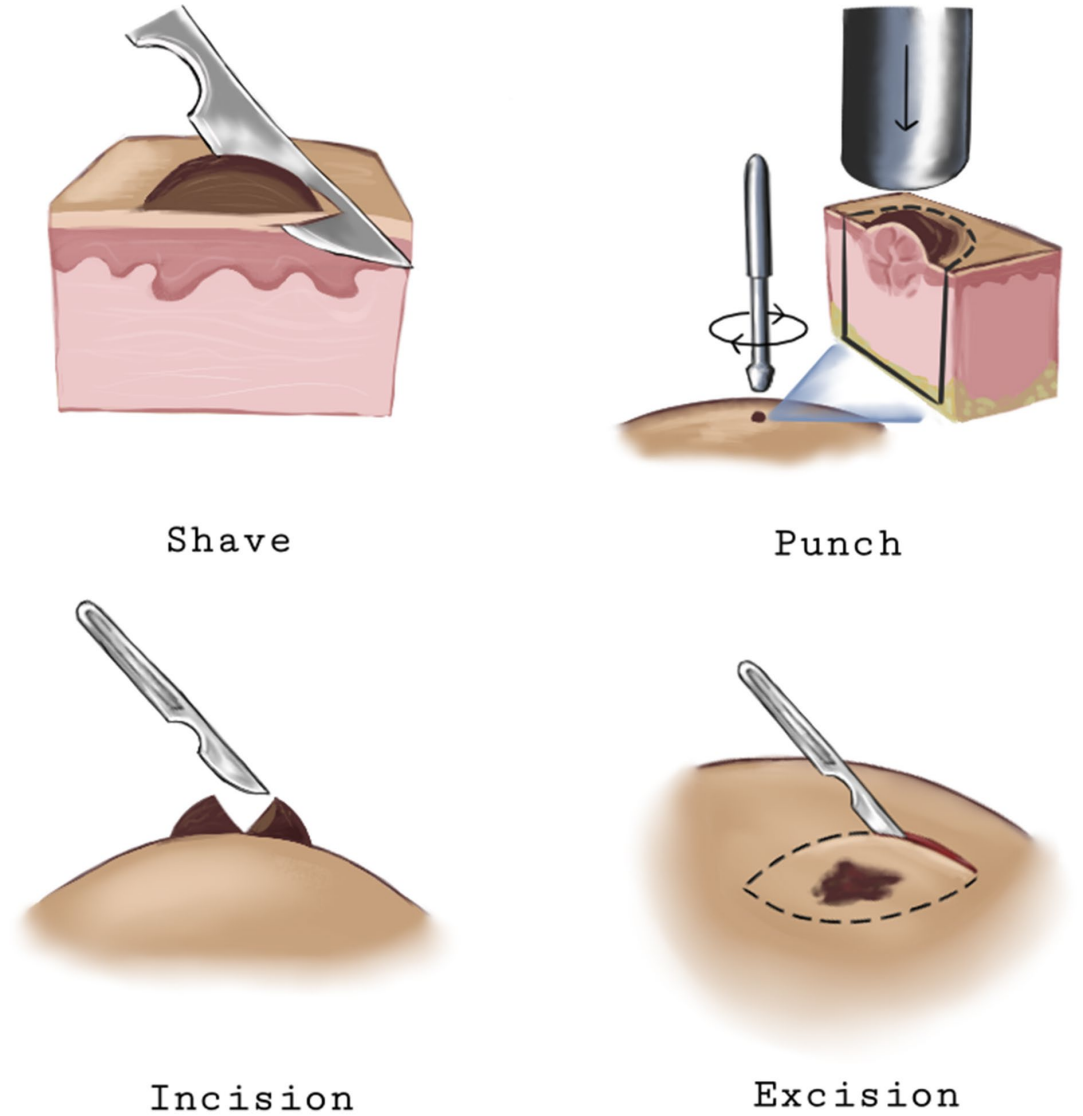
Illustration of different types of skin biopsies.

Several techniques are used to obtain skin biopsy specimens, each with its indications: Punch biopsy, where a circular blade is used to remove a cylindrical core of skin, including the epidermis, dermis, and superficial subcutis. It is commonly used to diagnose inflammatory and neoplastic skin conditions. Shave biopsy, where a scalpel or razor blade is used to shave off a superficial layer of skin. This method is suitable for lesions confined to the epidermis, such as warts and superficial basal cell carcinomas. Excisional biopsy, where the entire lesion is removed with a margin of normal skin. It is used for small, suspicious lesions where complete removal is necessary. An incisional biopsy, where a portion of the lesion is removed, is often used for larger lesions or when a diagnosis cannot be made with less invasive methods (21). Figure 1 shows the different types of skin biopsies.

### Artificial intelligence (AI) and convolutional neural networks (CNNs)

AI is a branch of computer science focused on creating systems capable of performing tasks that typically require human intelligence. These tasks include learning, reasoning, problem-solving, understanding language, and recognizing patterns (22). AI technologies, such as machine learning and neural networks, enable computers to learn from data and improve over time. AI is widely used in various fields, including healthcare, helping to automate processes, enhance decision-making, and solve complex problems. It enhances the ability to diagnose, treat, and manage diseases (23). In dermatology, AI, particularly through technologies such as CNNs, is used to analyze images of skin lesions, helping to detect conditions such as skin cancer with high accuracy (24). AI assists dermatologists by providing quick and precise analyses, reducing the time needed for diagnosis, and improving patient outcomes.

CNNs are a specialized type of AI designed to process and analyze visual data. It mimics the human brain’s way of recognizing patterns and features in images, making it particularly effective for tasks such as image classification, object detection, and facial recognition (25). CNNs are widely used in various fields, including healthcare, where they assist in diagnosing medical images, as well as in technology for applications such as self-driving cars and image search engines. Their ability to learn from large sets of labeled data allows them to identify complex patterns and provide accurate predictions. There are different types of CNN architectures available, such as NASNet-Large, AlexNet, Inception-ResNet-v2, Inception-v3, ResNet-50, SqueezeNet, and Vgg19, and (26, 27). In dermatology, CNNs are used to help diagnose skin conditions by examining images of skin lesions (28, 29). They can differentiate between various types of skin cancers such as melanoma and basal cell carcinoma, with high accuracy (30). By learning from thousands of labeled images, CNNs can assist dermatologists in making quicker and more accurate diagnoses, improving patient care and outcomes (31). However, a key concern with AI is bias caused by a lack of diverse skin tones in training data. Research shows that many AI systems are mainly trained on images of lighter skin, making them less accurate at diagnosing conditions in people with darker skin (32–34). A review, which identified that only 20% (14/70) about race and 10% (7/70) about skin color, reported that bias may contribute to higher rates of false positives and false negatives, thus affecting patient outcomes and contributing to healthcare differences (35). A recent systemic review has also reported that the majority of the studies reviewed were obtained from light skin and therefore provide insufficient evidence to comment on the overall accuracy of AI models for darker skin types. They concluded that the lack of diversity in studies is likely caused by the shortage of available datasets (36).

### AI techniques in skin biopsy

The excitement regarding AI in dermatology began in 2017 when a study compared the diagnostic performance of an AI-powered network with that of 21 board-certified dermatologists in evaluating biopsy-proven clinical images of benign and skin cancers. The findings showed that the AI system demonstrated diagnostic accuracy equal to that of human experts, achieving a level of competence comparable to that of experienced dermatologists (24).

Not widely applied but several studies have shown that direct image analysis is a reality for accurate classification of routine diagnoses for skin biopsies (37–40). In fact, one of the common uses of AI in skin biopsy is diagnosis. Basal cell carcinoma (BCC) showed a sensitivity of 98.23% and a specificity of 98.51% using CNNs from 1,255 whole-slide images (41). Another study revealed that CNNs achieved an accuracy of 99.5% for nodular BCC, 99.3% for dermal nevus (DN), and 100.0% for seborrheic keratosis (SK) (42). Another interesting finding regarding the AI is that CNNs, which were trained using 595 histopathologic images of melanomas and nevi, were classified by an expert dermato-histopathologist. When tested with an additional 100 images, the CNNs showed a discordance rate of only 19% compared to the histopathologist’s classifications (43). This rate is similar to the discordance between human pathologists, which is reported in the literature to be 25–26% (44, 45).

A total of 1,377 patches of healthy tissue and 2,141 patches of melanoma were assessed in the training/validation set, while 791 patches of healthy tissue and 1,122 patches of pathological tissue were evaluated in the test dataset. The CNN findings showed 95.7% sensitivity, 97.7% specificity, and 96.5% accuracy when compared with the dermatopathologist results (46). In a retrospective study, which reviewed 225,230 pathological patches cut from 79 formalin-fixed paraffin-embedded pathological slides from 73 patients (55 non-malignant eyelid nevus slides from 55 patients and 24 malignant melanoma slides from 18 patients), H&E stained whole-slide images (WSIs) and compared with 7 board-certified pathologists, the CNN findings showed 91.4% accuracy, 91% sensitivity, and 92.8% specificity (47).

In comparison with 95 human experts, of whom 62 were board-certified dermatologists, the CNNs, which were trained on 7,895 dermoscopic and 5,829 close-up images of lesions excised at a primary skin cancer clinic, showed a higher accuracy rate than those experts in diagnosis of common malignant cases such as basal cell carcinoma, actinic keratoses or Bowen disease, and squamous cell carcinoma or keratoacanthoma but did not reach the accuracy of human experts in rare malignant non-pigmented lesions such as amelanotic melanoma and benign non-pigmented lesions (48). It is important to note that amelanotic melanoma is not easy to diagnose, even for experts (49). Another systematic review of 39 studies for the detection of NMSC found that the AI overall diagnostic accuracy, in comparison with histopathologic diagnosis, was high and ranged from 72 to 100% (50). In a training set of 1,629 images (743 malignant lip, 886 benign lip diseases), the findings showed that CNNs were equivalent to the dermatologists and superior to the non-dermatologists in classifying malignancy (51). On a set of 1,417 images from 308 regions of interest on skin histopathology slides, where the presence or absence of basal cell carcinoma needs to be determined, the findings showed that deep learning architectures had a 91.4% accuracy in comparison with histopathology (52).

Another important use of AI in dermatology is with onychomycosis, which is best demonstrated by the periodic-acid-Schiff (PAS) staining method in comparison with other methods such as direct microscopy using potassium hydroxide staining, fluorescence optical preparation, and culture (53, 54). PAS has high specificity, is not expensive, and the detection of tinea is high if present in a high number (55). However, if the fungi are present in a small number, histopathologic detection is time-consuming and the risk of missing fungi is high (56). Subsequently, this might result in a delay in diagnosis and repeating preparation and analysis (57). The literature shows that the sensitivity of detecting fungi using histopathologic evaluation has been reported to range between 80 and 85% (58). A study, that used CNNs with 664 corresponding H&E- and PAS-stained histologic whole-slide images (WSIs) of human nail plates from four different laboratories, showed a sensitivity and specificity of 93 and 77%, respectively. Their study demonstrated comparable sensitivity to that of the 11 board-certified dermatopathologists (56). Another similar study reported that CNNs showed 94.1% sensitivity and 98% specificity, for a dataset of 528 whole-slide images of nail samples for onychomycosis (59). Another interesting finding of AI with microorganisms is that the CNN model in a dataset of 1,819 thick smear images from 150 patients showed effectiveness in discriminating between positive (parasitic) and negative image patches with 93.46% accuracy, 92.59% sensitivity, 94.33% specificity, 94.25% precision, and 92.74% negative predictive value (60). A recent systemic review on the use of AI in skin disease diagnosis in primary care settings showed that CNN has a sensitivity ranging from 58 to 96.1%, with accuracies varying from 41 to 93% (61). Other studies have used AI in research in dermatopathology (62, 63). These findings highlight the AI potential to enhance diagnostic accuracy and efficiency in dermatopathology.

Artificial intelligence (AI) applications in frozen section analysis are advancing to assist pathologists in rapidly evaluating slides, identifying cancerous cells, and distinguishing them from healthy tissue, which enhances diagnostic speed and accuracy in the operating room setting. Several studies using AI and deep learning models have been valuable tools in enhancing the assessment of MMS slides for skin cancer detection with high sensitivity and specificity rates (64, 65). A review of selected recent articles on the application of artificial intelligence in diagnosing and managing skin diseases is shown in Table 1.

**Table 1:** Review of selected recent articles on the application of artificial intelligence in diagnosing and managing skin diseases.

Authors	Study titles	AI type	Publication year
Marsden et al. (84)	Accuracy of artificial intelligence as a medical device as part of a UK-based skin cancer teledermatology service	CNN: Artificial intelligence medical device	2024
Liu et al. (85)	Predicting skin cancer risk from facial images with an explainable artificial intelligence (XAI) based approach: a proof-of-concept study	Explainable artificial intelligence	2024
Tan et al. (86)	Development and validation of a deep learning model for improving detection of non-melanoma skin cancers treated with Mohs micrographic surgery	CNN	2024
Li et al. (87)	Deep learning approach to classify cutaneous melanoma in a whole-slide image	EfficientNetB1 CNN	2023
Cozzolino et al. (88)	Machine learning to predict overall short-term mortality in cutaneous melanoma	Logistic regression classifier, Support vector machine, Random forest, Gradient boosting, k-nearest neighbors, Deep neural network	2023
Aung et al. (89)	Objective assessment of tumor-infiltrating lymphocytes as a prognostic marker in melanoma using machine learning algorithms	NN192	2022
Brodsky et al. (90)	Performance of automated classification of diagnostic entities in dermatopathology validated on multisite data representing the real-world variability of pathology workload	CNN	2022
Dika et al. (91)	Advantages of manual and automatic computer-aided compared to traditional histopathologic diagnosis of melanoma: A pilot study	CNN	2022
Couetil et al. (92)	Predicting melanoma survival and metastasis with interpretable histopathologic features and machine learning models	CNN	2022
Figueroa-Silva et al. (93)	Machine learning techniques in predicting BRAF mutation status in cutaneous melanoma from clinical and histopathologic features	Random forest, Support vector machine, and Extreme gradient boosting	2022
Li et al. (94)	Application of deep learning on the prognosis of cutaneous melanoma based on full-scan pathology images	Deep learning (VGG-19), machine learning (SVM)	2022
Comes et al. (95)	A deep learning model based on whole-slide images to predict disease-free survival in cutaneous melanoma patients	CNNs: ResSVM, DenseSVM, InceptionSVM	2022
Mund et al. (96)	Deep visual proteomics defines single-cell identity and heterogeneity	Deep visual proteomics and deep learning, machine learning	2022
Cazzato et al. (97)	Dermatopathology of malignant melanoma in the era of artificial intelligence: a single institutional experience	Fast random forest algorithm	2022
Wang et al. (98)	Self-supervised learning mechanism for identification of eyelid malignant melanoma in pathologic slides with limited annotation	Self-supervised learning CNN	2022
Kriegsmann et al. (99)	Deep learning for the detection of anatomical tissue structures and neoplasms of the skin on scanned histopathologic tissue sections	EfficientNetV2 architecture	2022
Nielsen et al. (100)	Computer-assisted annotation of digital H&E/SOX10 dual stains generates a high-performing convolutional neural network for calculating tumor burden in H&E-stained cutaneous melanoma	CNN	2022
Sturm et al. (101)	Computer-aided assessment of melanocytic lesions by means of a mitosis algorithm	CNN: mitosis algorithm	2022
Wan et al. (102)	Prediction of early-stage melanoma recurrence using clinical and histopathologic features	Support vector machine, Gradient boosting, Random forest, Logistic regression, Multilayer perceptron	2022
Snyder et al. (103)	Histologic screening of malignant melanoma, spitz, dermal, and junctional melanocytic nevi using a deep learning model	CNN standard ResNet-50	2022
Doeleman et al. (104)	Artificial intelligence-assisted probability scoring for differentiation of early mycosis fungoides and benign inflammatory dermatoses on H&E-stained pathology slides of skin biopsies	Clustering-constrained Attention Multiple Instance Learning (CLAM)	2022

CNN, convolutional neural network.

### Benefits of AI in skin biopsy

AI assists in the interpretation of biopsy samples. AI serves as a valuable tool for pathologists, offering second opinions and highlighting areas of concern within biopsy samples (40). AI can reduce diagnostic errors by providing consistent and objective analysis, minimizing inter- and intra-observer variability (66). AI can process large volumes of biopsy samples quickly, reducing turnaround times and enabling faster diagnosis and treatment initiation. AI can be used to predict disease outcomes and responses to treatment based on biopsy findings and other patient data, aiding in personalized medicine and tailored therapeutic strategies (67).

### Future directions for skin biopsy

As technology advances, there is a growing potential to enhance the diagnostic abilities of skin biopsy through innovative techniques and tools. Future developments aim to improve the precision, efficiency, and comprehensiveness of skin biopsies, finally benefiting patient care. Regarding innovations in biopsy techniques, there have been significant advancements in minimally invasive procedures and liquid biopsy methods. Microneedle biopsy involves the use of microneedle arrays to collect small amounts of tissue or interstitial fluid with less discomfort and scarring for patients (68, 69). Laser-assisted biopsy uses lasers to precisely target and excise tissue samples, minimizing damage to surrounding areas and enhancing accuracy while reducing healing times (70). Additionally, research into liquid biopsy focuses on identifying circulating biomarkers, such as cell-free DNA, RNA, and proteins, which can provide diagnostic information from a blood sample and potentially reduce the need for traditional tissue biopsies (71). Due to advances in multi-omics such as genomics, transcriptomics, and in genomic analyses such as next-generation sequencing, the identification of many gene mutations has been explored, as with BRAF, NRAS, and c-KIT in melanoma (72–74). Regarding personalized medicine and precision dermatology genomic and molecular profiling by integrating these data with skin biopsy results to gain a comprehensive understanding of individual patient conditions. This approach can guide targeted therapies and improve treatment outcomes (75, 76). Pharmacogenomics, the study of how genes affect a person’s response to drugs, is increasingly being used in skin biopsy analysis to modify treatments and improve outcomes. This approach allows for personalized medicine, where genomic and molecular profiling of biopsy samples can reveal specific genetic variations that influence drug efficacy and safety (77). For instance, understanding a patient’s unique genetic makeup can help dermatologists select the most effective therapies and minimize adverse effects, optimizing the treatment of skin cancers and other dermatological conditions (78, 79). As technology evolves, the integration of pharmacogenomics with skin biopsies holds promise for more precise and effective dermatological care.

### Limitations of AI in skin biopsies

Despite the prevalence of skin lesions, scientists face challenges in obtaining annotated training and skin images as skin disease images are still insufficient (80). One critical challenge with CNNs is their need for a large amount of data; the quality and size of the image dataset are essential for effective CNN training and validation (81). Another important drawback is that experts in computer science, biomedical, and medicine are insufficient (82). Furthermore, gaining acceptance from healthcare professionals requires addressing concerns about reliability and transparency. Another major issue with AI is determining who is responsible for any diagnostic errors made by the AI. As well as other moral and ethical issues related to the use of AI. In addition, there are many kinds of skin diseases (83). It might be difficult for AI to identify all specific skin diseases. Furthermore, as AI models trained mostly on lighter skin tones may have lower accuracy for darker skin, this might raise concerns about diagnostic differences (36).

## Conclusion

Histopathological diagnosis of skin biopsy is still the gold standard method for skin diseases. However, the integration of traditional histopathological techniques such as light microscopy and immunofluorescence with advanced technologies such as artificial intelligence and convolutional neural networks enhances diagnostic accuracy and efficiency. Innovations in biopsy techniques, including minimally invasive procedures and liquid biopsies, should further improve patient outcomes by reducing discomfort and providing comprehensive diagnostic information. The application of pharmacogenomics in skin biopsy analysis facilitates personalized medicine by modifying treatments based on individual genetic profiles. Despite the advancements, challenges remain, in particular, in obtaining high-quality annotated images for training AI models and addressing ethical and responsibility concerns associated with AI diagnostics. Overall, the review highlights the evolution and future potential of skin biopsy practices in improving dermatological care.
